# From detached to alarmed: How eco-emotion profiles predict concern and sacrifice for the planet

**DOI:** 10.1371/journal.pone.0325916

**Published:** 2025-06-17

**Authors:** Andreas K Jäger, Donald W Hine

**Affiliations:** School of Psychology, Speech and Hearing, University of Canterbury, Christchurch, New Zealand; Vrije Universiteit Brussel, BELGIUM

## Abstract

The rapid degradation of the environment is one of our greatest challenges in the 21^st^ century. To avoid the worst consequences, human behavior change is required. The current study investigated how feelings about environmental problems (eco-emotions) predict concern for the natural environment and willingness to make sacrifices for it. Using a cross-sectional online sample of 286 New Zealand residents, latent profile analysis identified three profile groups with distinct patterns of eco-emotions: emotionally-detached (40%), emotionally-ambivalent (34%), and empathic-alarmed (26%). Validation analyses revealed that members of the empathic-alarmed segment reported significantly higher levels of environmental concern and willingness to make sacrifices for the environment than members of the emotionally-ambivalent segment, who in turn expressed greater concern and willingness to sacrifice than members of the emotionally-detached segment. Findings from this study suggest that inducing a combination of negative eco-emotions with compassion may be effective for promoting environmental concern and pro-environmental sacrifice.

## Introduction

Emotions play an important role in shaping attitudes, threat appraisals, and decision-making processes, including those related to environmental issues such as climate change and sustainability. To avoid significant and potentially irreversible disruptions to the natural environment and human societies, scientists advise humans to live within planetary boundaries: the limits within which humanity can safely operate to maintain a stable and resilient earth system [1,2]. Recent research, however, indicates that we have already exceeded safe limits of six of nine boundaries identified by Rockström and colleagues [1]: climate change, novel entities, biogeochemical flows, land-system change, freshwater use, and biosphere integrity [1–4]. Despite these well-documented environmental threats and the need for human behavior change, the impact of humans on the planet has consistently trended upwards. Early in the 1970s, humanity’s annual demand for biological resources only marginally exceeded the amount the Earth ecosystem could regenerate. By 2022 this demand exceeded the ecosystem regeneration capacity by at least 70%. Reversing this trend constitutes one of humanity’s greatest challenges. In the current study, we explore how feelings about environmental problems (eco-emotions) predict concern for the natural environment and willingness to make sacrifices to save it.

### Eco-emotions

In response to the growing number and intensity of environmental challenges, researchers in environmental psychology and behavioral economics have become increasingly interested in situational and personal factors that influence pro-environmental behavior [PEB; 5–7]. The well-established link between emotions and behavior in other domains [8–13] has prompted environmental researchers to explore the relevance of emotional responses to PEB.

Emotions are complex psychological and physiological responses to personally meaningful objects and events, which can include feelings, cognitive appraisals, and expressed behavioral tendencies [14]. Eco-emotions tend to be defined more narrowly, generally focusing on subjective feelings toward ecological issues, such as anxiety, guilt, anger, and hope [15]. Eco-emotions are considered to play an important role in shaping cognitive appraisals such as environmental concern and motivating PEB [16–18].

Several recent international studies have investigated the prevalence of eco-emotions and their relevance to PEB [19–21]. In a multinational study with 10,000 participants, Hickman and colleagues [19] found that many young people experienced largely negative eco-emotions, which are associated with climate distress and a negative functional impact on mental health. Fear (67%) was the most commonly reported emotion when thinking about climate change, followed by anxiety (61%), worry (60%), anger (57%), and guilt (50%), while only a small proportion of participants felt optimistic (30%) and indifferent (29%).

Negative eco-emotions are not only common but have also increased over time. Leviston and colleagues [20] found that between 2010 and 2013 Australians became more ashamed and guilty when thinking about climate change and less hopeful. Leiserowitz and colleagues [21] found a similar trend in the US, with two thirds of Americans being worried about climate change, which is an increase of approximately 10% within five years (2014–2019). Furthermore, the number of Americans feeling very worried (30%) about climate change tripled during this period. Overall, research suggests that people around the world exhibit a broad range of emotional responses to environmental challenges, including negative emotions such as worry, fear, and shame, and to a lesser extent positive emotions such as hope and optimism. While we, and others, refer to emotions as “positive” (e.g., hope, happiness) or “negative” (e.g., anger, guilt) based on their subjective valence, it is important to note that emotions are not inherently good or bad. From an evolutionary perspective, most emotions – when experienced within a normal range – are functional and adaptive, serving to guide attention, shaping behavior, and facilitating social coordination [13,22]. Thus, the distinction between positive and negative emotions in this paper reflects valence rather than value.

### Eco-emotions and PEB

Emotions play a key role in shaping attitudes and threat appraisals, both of which have been shown to be important determinants of behavior. Ajzen’s theory of planned behavior (TPB; 23) posits that emotions may help individuals to generate positive or negative attitudes, which influence intentions and behavior. Similarly, Slovic and colleagues’ affect heuristic model [24] suggests that people’s affective responses (general feelings of goodness or badness) towards an object, event, or behavior can influence risk appraisals, which in turn can increase or decrease the likelihood of future actions. Emotions may play a particularly important role for acting environmentally-friendly. Theorists argue that emotions may facilitate PEB by serving as a mechanism to concretize or reduce the psychological distance of abstract environmental problems like climate change, for which the most extreme impacts are often perceived to be highly uncertain, likely to take place far in the future, in distant locations, and impacting dissimilar others [25,26]. This concretization process can enhance the salience of environmental problems, feelings of perceived personal vulnerability, and empathy for affected others, all of which may further enhance PEB [16].

There is considerable evidence to suggest that specific emotions are predictive of decisions to engage or not engage in PEB: literature on fear appeals, for instance, indicates that fear can elicit behaviors to reduce external threats, particularly when accompanied by information that boosts self-efficacy [27,28]. Being worried about environmental problems, on the other hand, has been suggested to cause a feeling of personal responsibility and thereby lead to PEB [29]. Other eco-emotions such as pride [30], guilt [28,30,31], hope [32,33], and anger and depression were also found to increase the likelihood to engage in climate activism [34,35], while experiencing eco-anxiety has shown mixed results [34,35], with some studies suggesting that increased anxiety was associated with lower levels of PEB, and other suggesting the opposite. Stanley and colleagues [34] reported that eco-anxiety was associated with lower levels of PEB, while a multinational study [35] across 32 countries found that experiencing anxiety towards climate change was in most countries associated with higher levels of PEB and environmental activism. In general, previous studies suggest that a range of eco-emotions predict PEB, although the direction of these associations is sometimes inconsistent.

To date, most studies on emotions and PEB have examined the role of how specific emotions in isolation relate to human responses to environmental problems [28–31,34–36]. However, it is important to recognize that eco-emotions often co-occur, and that the overall pattern of emotional responses, as opposed to the experience of individual emotions, may help us better understand why people respond or fail to respond to environmental threats. Using a nationally representative US sample, Smith and Leiserowitz [17] found that a combination of five eco-emotions (worry, hope, interest, guilt, and disgust) explained 50% of the variance in climate change policy support, explaining pro-environmental tendencies better than worldviews and socio-demographic variables. These findings highlight the importance of eco-emotions for sustainable behavior, but also suggest that multiple eco-emotions may combine in complex ways to influence PEB. Further evidence for the interplay between emotions has been provided by Ojala [32], who found that hope predicted PEB, but only for individuals who were also worried about environmental problems. One explanation for this may be that when people are hopeful, but not worried, hope is based on denial, but when people are hopeful and worried, they experience constructive hope which increases the motivation to practice PEB [33]. Wang and colleagues [18] further extended our understanding of the complex interplay of eco-emotions by evaluating whether a combination of a wider range of eco-emotions (13 emotions in total) predicted PEB. Using hierarchical cluster analysis, they identified four audience segments with distinct sets of eco-emotions which were characterized as having either strong negative emotions (scoring high on negative emotions such as anger and fear, but low on positive emotions like hope), weak negative emotions (scored similar to those with strong negative emotions, but with lower intensity), no emotions (experienced low levels of both negative and positive emotions), and ambivalent emotions (experienced both positive and negative emotions). Members of the strong negative emotions segment reported significantly higher levels of support for climate friendly policies and environmental funding compared to members of the weak negative emotions and ambivalent-emotions segments. Members of the no emotions segment exhibited the lowest levels of policy and funding support. Given the multidimensional and co-occurring nature of eco-emotions, a person-centered segmentation approach such as latent profile analysis (LPA) offers a valuable means of identifying distinct emotional profiles. This method allows us to move beyond single-emotion predictors and explore how patterns of affective experiences predict pro-environmental concern and behavior.

Building on the TPB [23] and the affect heuristic model [24], this study considers how eco-emotions shape motivational pathways to PEB. From a TPB perspective, emotions can influence attitudes toward sustainability and strengthen intentions to act, while the affect heuristic suggests that emotional responses to environmental issues influence perceived risk and concern. Together, these frameworks support the proposition that specific combinations of eco-emotions may increase environmental concern and willingness to sacrifice for the planet.

### The current study

The current study seeks to deepen our understanding of which eco-emotions tend to co-occur and how patterns of emotions predict environmental concern and willingness to sacrifice. Our study extends Wang and colleagues’ [18] work both conceptually and methodologically. First, whereas Wang and colleagues focused on eco-emotions related to climate change, we focused more broadly on emotional responses towards environmental problems. Second, while they investigated how members of each eco-emotions segment differed in terms of their support for climate-friendly policies and funding, we analyzed how our identified emotions segments predict environmental concern and willingness to make a broad range of personal sacrifices related to climate change mitigation but also more generally towards environmental sustainability. Measuring willingness to sacrifice considers that pro-environmental decisions are often a trade-off between doing what is best for oneself versus what is best for the environment [37]. In contrast to traditional behavioral measures, willingness to sacrifice is a more diverse measure as it can cover a wide range of intentions for various PEBs rather than assessing context specific behaviors. Third, whereas Wang and colleagues created eco-emotion segments based on a relatively restricted set of 13 emotions, we assessed a larger group of 23 discrete emotions to create a segmentation solution. Our selection of eco-emotions includes different aspects of unhappy emotions (e.g., sad, hurt, grief), fear-related emotions (i.e., anxious, afraid, worried), positive emotions (i.e., optimistic, happy), and an empathic emotion (i.e., compassion) which were previously not assessed in detail by Wang and colleagues. Using a larger set of eco-emotions allowed us to evaluate the possible effects of emotions on environmental concern and willingness to sacrifice in a more complete and nuanced manner.

Based on Wang and colleagues’ [18] findings, we hypothesized that there would be at least three eco-emotion segments in our sample: negative eco-emotions, no emotions, and ambivalent emotions (a mix of positive and negative eco-emotions). We also predicted that the identified segments would differ significantly in their environmental concern and willingness to sacrifice, with those groups feeling stronger negative eco-emotions exhibiting the highest levels of environmental concern and willingness to sacrifice for the natural environment, and those with no eco-emotions exhibiting the lowest levels.

## Method

### Participants

The study was conducted in August 2022 and used a Cint online sample of New Zealanders aged 18 years and above. Participants were recruited by Cint using proprietary algorithms to ensure our sample was roughly representative of the New Zealand population in terms of age, gender, and ethnicity. After completing the survey, participants received a small remuneration for their time. Cint is one of the largest digital survey-based providers with access to over 500,000 panelists [38]. To ensure survey data quality, Cint [39] uses a detection system to prevent duplicates and fraud, and suppliers adhere to industry standards [40]. Of the 405 New Zealand residents recruited, 119 participants were excluded from the survey as they either did not complete the survey or did not pass all three attention checks. The final sample consisted of 286 participants aged 18–89 years (*M *= 43.13, *SD* = 17.53; *Median* = 40.00; S1 Table). Compared to 2018 census data [41], participants in the current sample were slightly older (median age in current study: 40.00 years; census: 37.40 years), more educated (participants holding an undergraduate degree or higher in current study: 41%; census: 25%), and consisted of more females (current study: 66%; census: 51%). In terms of ethnicity, the current sample resembled the New Zealand population (current study: 67% European, 17% Māori; census: 68% European, 15% Māori). Most participants lived in cities or suburban areas (64%), while 36% lived in rural residential, semi-rural, and rural areas.

### Procedure and measures

The study was reviewed and approved by the Human Research Ethics Committee at the University of Canterbury. An online survey was created with the survey platform Qualtrics [42] and distributed to a Cint-sourced online panel. At the beginning of the survey, participants were informed about the purpose of the study and that responses would be anonymous. Moreover, written informed consent was obtained, participants were told that they could choose to withdraw at any time from the survey, and a helpline was provided in case any of the questions caused them distress.

After answering questions about their socio-demographic background, participants were asked how they feel towards environmental problems by rating 23 different types of eco-emotions (sad, hurt, angry, disgusted, frustrated, afraid, anxious, worried, ashamed, guilty, despair, depressed, grief, helpless, powerless, indifferent, bored, confused, doubtful, optimistic, hopeful, happy, compassionate). All emotion items were derived from previous research by Hickman and colleagues [19] and Pihkala [15] and were measured on a 5-point Likert scale (1 = never to 5 = almost always).

Next, participants’ environmental concern was measured using an amended version of the *Concern for Climate Change* scale applied by Jones and colleagues [26]. The scale consisted of seven items, of which one item was reverse coded (“I am not overly concerned about environmental problems such as climate change as I think the impacts are probably exaggerated”). All items were measured on a 5-point Likert scale (1 = strongly disagree to 5 = strongly agree). Internal consistency for this scale was very high (Cronbach’s α = .92).

Finally, participants’ willingness to make sacrifices for the natural environment was assessed with a newly developed 24-item scale. As measuring PEB in real world settings is often difficult, we employed a measure of PEB intentions based on the concept of willingness to sacrifice. Willingness to sacrifice, which is “the extent to which individuals’ decisions will take into account the well-being of the environment, even at the expense of immediate self-interest, effort, or costs” (37 p259), has been found to be one of the strongest predictors of PEB, over and above knowledge, concern, and socio-economic characteristics [37]. Although several willingness to sacrifice scales exist [36,37], their focus is quite narrow. We developed a new scale to assess a broader range of sustainable behaviors related to transportation, food, electricity, and recycling, which were not assessed by existing scales. All items were measured on a 5-point Likert scale (1 = strongly disagree to 5 = strongly agree). While exploratory factory analysis, using principal axis factoring, revealed four eigenvalues above one, the scree plot clearly indicated a one-factor solution. All solutions from one to four were subjected to promax rotations. Solutions two to four exhibited a high number of cross-loadings, and several items did not load strongly on any factor. Consistent with the scree plot, the one-factor solution was most interpretable and therefore retained to construct a unidimensional measure of willingness to sacrifice. The average of the 24 items was calculated to create a single score. Internal consistency for this scale was very high (Cronbach’s α = .92). Details of the questionnaire are shown in S2 Appendix.

### Statistical methods

To analyze the data [43], SPSS 28 [44] was used to conduct descriptive statistical analysis and assess bivariate correlations among all study variables. LPA was then performed with MPlus 8 [45] to segment the respondents into homogenous groups based on their eco-emotions. A series of MANOVAs and ANOVAs were conducted using SPSS 28 to determine if segment membership predicted differences in a range of outcome variables, including demographics, environmental concern, and willingness to sacrifice.

## Results

### Descriptive statistics and bivariate correlations

Means, standard deviations, and bivariate correlations between all variables are presented in Table 1. Most eco-emotions were significantly positively correlated with environmental concern, apart from indifferent and bored which were significantly negatively correlated with environmental concern, and optimistic, hopeful, and happy which did not correlate with environmental concern. Most eco-emotions were significantly positively correlated with willingness to sacrifice, apart from bored which was negatively correlated. Indifferent and happy were uncorrelated with willingness to sacrifice.

**Table 1:** Descriptive statistics and correlation matrix of study variables.

	Variable	*M*	*SD*	1	2	3	4	5	6	7	8	9	10	11	12	13	14	15	16	17	18	19	20	21	22	23	24	25
1	Sad	2.80	1.31	—																								
2	Helpless	2.65	1.33	.60^**^	—																							
3	Anxious	2.43	1.27	.65^**^	.57^**^	—																						
4	Afraid	2.42	1.28	.69^**^	.59^**^	.74^**^	—																					
5	Optimistic	2.20	1.12	.02	−.09	.01	.04	—																				
6	Angry	2.51	1.34	.77^**^	.56^**^	.67^**^	.71^**^	.02	—																			
7	Guilty	2.31	1.29	.63^**^	.56^**^	.61^**^	.60^**^	−.01	.65^**^	—																		
8	Ashamed	2.33	1.30	.69^**^	.51^**^	.60^**^	.62^**^	.02	.66^**^	.73^**^	—																	
9	Hurt	2.21	1.27	.64^**^	.53^**^	.60^**^	.66^**^	.09	.70^**^	.57^**^	.60^**^	—																
10	Depressed	2.07	1.20	.60^**^	.49^**^	.57^**^	.50^**^	.03	.57^**^	.50^**^	.53^**^	.56^**^	—															
11	Despair	2.30	1.23	.67^**^	.56^**^	.65^**^	.62^**^	.02	.66^**^	.61^**^	.62^**^	.66^**^	.59^**^	—														
12	Grief	2.13	1.15	.61^**^	.50^**^	.54^**^	.61^**^	.11	.64^**^	.52^**^	.58^**^	.63^**^	.54^**^	.62^**^	—													
13	Powerless	2.90	1.35	.46^**^	.74^**^	.47^**^	.50^**^	−.06	.45^**^	.47^**^	.43^**^	.46^**^	.42^**^	.45^**^	.43^**^	—												
14	Indifferent	1.92	1.06	.06	.14^*^	.15^**^	.16^**^	.15^**^	.12^*^	.14^*^	.09	.24^**^	.19^**^	.11	.19^**^	.24^**^	—											
15	Hopeful	2.34	1.13	.06	−.09	.09	.05	.60^**^	.02	.04	.10	.11	.10	.06	.14^*^	−.03	.19^**^	—										
16	Disgusted	2.64	1.38	.70^**^	.58^**^	.57^**^	.60^**^	.02	.74^**^	.63^**^	.63^**^	.63^**^	.51^**^	.64^**^	.58^**^	.49^**^	.11	.06	—									
17	Happy	1.75	1.07	−.14^*^	−.17^**^	−.05	−.05	.41^**^	−.12^*^	−.12^*^	−.10	.01	−.05	−.10	−.04	−.18^**^	.26^**^	.47^**^	−.10	—								
18	Compassionate	2.81	1.18	.44^**^	.24^**^	.35^**^	.34^**^	.22^**^	.37^**^	.33^**^	.33^**^	.37^**^	.32^**^	.38^**^	.39^**^	.17^**^	−.03	.39^**^	.38^**^	.23^**^	—							
19	Confused	2.12	1.13	.33^**^	.45^**^	.48^**^	.49^**^	.15^*^	.42^**^	.45^**^	.42^**^	.40^**^	.40^**^	.47^**^	.44^**^	.38^**^	.29^**^	.11	.40^**^	.08	.15^*^	—						
20	Bored	1.64	1.00	.05	.12	.13^*^	.17^**^	.17^**^	.08	.09	.11	.14^*^	.24^**^	.10	.22^**^	.13^*^	.54^**^	.19^**^	.13^*^	.26^**^	.02	.27^**^	—					
21	Doubtful	2.28	1.17	.41^**^	.46^**^	.45^**^	.47^**^	.09	.43^**^	.42^**^	.45^**^	.51^**^	.39^**^	.42^**^	.47^**^	.47^**^	.26^**^	.10	.45^**^	.09	.22^**^	.36^**^	.24^**^	—				
	Variable	*M*	*SD*	1	2	3	4	5	6	7	8	9	10	11	12	13	14	15	16	17	18	19	20	21	22	23	24	25
22	Worried	2.81	1.28	.74^**^	.60^**^	.72^**^	.72^**^	.06	.72^**^	.61^**^	.63^**^	.63^**^	.58^**^	.69^**^	.62^**^	.49^**^	.12^*^	.10	.63^**^	−.12^*^	.37^**^	.47^**^	.06	.47^**^	—			
23	Frustrated	2.77	1.32	.73^**^	.59^**^	.66^**^	.64^**^	−.02	.77^**^	.60^**^	.61^**^	.61^**^	.51^**^	.64^**^	.56^**^	.50^**^	.05	.06	.76^**^	−.11	.41^**^	.40^**^	.03	.45^**^	.72^**^	—		
24	Environmental Concern	3.66	.95	.58^**^	.41^**^	.52^**^	.50^**^	−.03	.53^**^	.45^**^	.49^**^	.44^**^	.35^**^	.50^**^	.42^**^	.25^**^	−.20^**^	.08	.47^**^	−.10	.39^**^	.17^**^	−.17^**^	.22^**^	.57^**^	.53^**^	—	
25	Willingness to Sacrifice	3.26	.76	.40^**^	.29^**^	.42^**^	.34^**^	.12^*^	.42^**^	.34^**^	.34^**^	.35^**^	.29^**^	.41^**^	.36^**^	.16^**^	−.09	.20^**^	.40^**^	.02	.44^**^	.20^**^	−.12^*^	.19^**^	.39^**^	.39^**^	.59^**^	—
																												

*N *= 286. All items were measured on a 5-point Likert scale (1 = minimum to 5 = maximum). * *p* < .05. ** *p* < .01.

### Eco-emotions segmentation

To better understand how distinct patterns of eco-emotions relate to environmental concern and willingness to sacrifice, we conducted an LPA using MPlus 8 [45]. The LPA enabled us to identify the number and nature of distinct eco-emotion segments within our sample, with subsequent analyses evaluating the extent to which the segments differed on a range of variables, including environmental concern and willingness to sacrifice. To assess the model fit, entropy (measure of classification uncertainty; 46), Akaike’s information criterion (AIC), and the Lo-Mendell-Rubin likelihood ratio test (LMR) were used. In general, the model with the smallest AIC values and the highest entropy (ranging from 0 to 1, with higher values indicating higher classification certainty) is the best fitting model [46–49]. The LMR is a test comparing the likelihood of one model with *k* segments to a model with *k*-1 variables [46]. A significant result of the LMR indicates that a model with *k* segments fits the data better than a model with *k*-1 segments. Model fit indices for two- to five-segment solutions are shown in Table 2.

**Table 2:** Model fit indices for two to five segment solutions for eco-emotions.

Segment Solution	AIC	BIC	Entropy	LMR	*p*
2	18824.74	19080.66	.97	2563.33	<.001
**3**	**18234.13**	**18577.79**	**.95**	**633.94**	**.001**
4	18807.88	18439.29	.93	272.24	.600
5	17909.01	18428.16	.94	145.79	.283

AIC = Akaike’s information criterion. BIC = Bayesian information criteria. LMR = Lo-Mendel-Rubin likelihood ratio test. Lower AIC and BIC values suggest a better model fit. Higher entropy indicates higher classification certainty. A significant LMR test indicates that a model with *k* segments fits the data significantly better than a model with *k*-1 segments.

**Fig 1:**
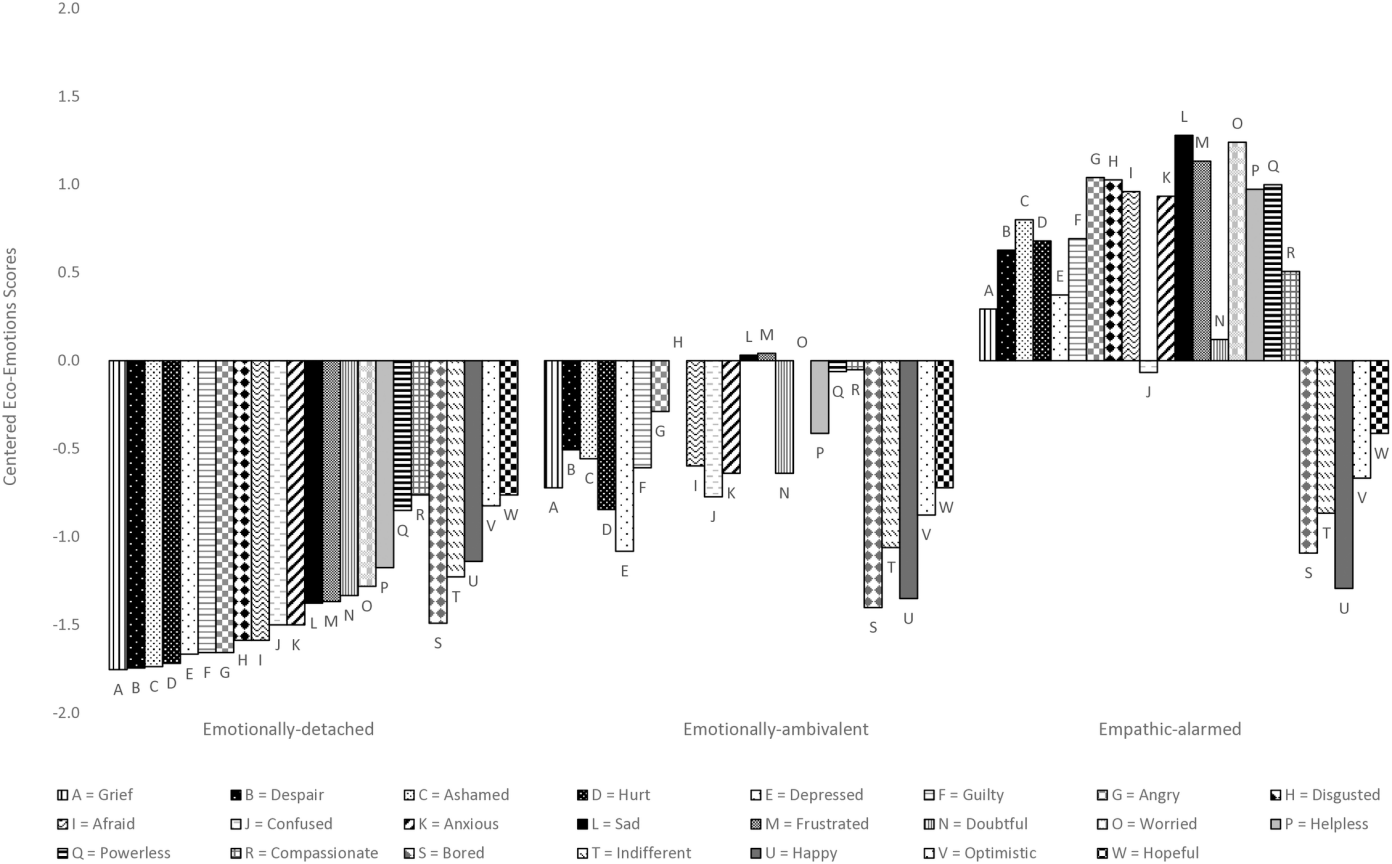
Eco-emotion segmentation solution. *Notes.* Participants were asked to rate how environmental problems made them feel. *N *= 286. Emotionally-detached, *n* = 114. Emotionally-ambivalent, *n* = 97. Empathic-alarmed, *n *= 75. Scores were centered and ranged from −2 (strongly disagree) to +2 (strongly agree).

A classification consisting of three segments fit the data significantly better than a two-segment solution, and a four-segment solution did not fit the data significantly better than a three-segment solution. The three-segment solution had a lower BIC value than the two-segment solution, indicating a better fit of the data. Entropy for the three-segment solution was high (.95), indicating high classification certainty [46]. Moreover, the three-segment solution was more interpretable than the other segment solutions. As such, the three-segment solution was retained and analyzed. The three segments were labelled emotionally-detached, emotionally-ambivalent, and empathic-alarmed. Fig 1 presents the deviation from the scale midpoint for all eco-emotions of the three identified segments.

The emotionally-detached segment, which comprised 40% of all respondents, scored well below the mid-point on all eco-emotions assessed in the study. Members of the second largest segment, the emotionally-ambivalent (34%), scored below the scale midpoint for all emotions, apart from disgust and worry, which were at the scale midpoint, and sadness and frustration, which were slightly above the scale midpoint. The emotionally-ambivalent had significantly higher levels of all negative eco-emotions (i.e., grief, despair, ashamed, hurt, depressed, guilty, angry, disgusted, afraid, confused, anxious, sad, frustrated, doubtful, worried, helpless, powerless) and compassion than the emotionally-detached (Table 3). However, these two segments did not differ significantly in their positive (i.e., optimistic, hopeful, happy) and apathetic (i.e., indifferent, bored) eco-emotions. The third segment, the empathic-alarmed, was the smallest segment (26%) and scored above the scale midpoint for most eco-emotions, with sadness, worry, and frustration being the highest rated eco-emotions. The empathic-alarmed segment scored below the scale midpoint for optimism, indifference, hope, happiness, confusion, and boredom. Compared to the emotionally-ambivalent and emotionally-detached, the empathic-alarmed had significantly higher levels of all negative eco-emotions and compassion, but did not differ significantly in their positive and apathetic eco-emotions.

**Table 3:** Mean scores, standard deviations, and mean differences for individual eco-emotions across the three eco-emotion segments.

Segment Variables	*Emotionally-detached* (*n *= 114)		*Emotionally-ambivalent* (*n *= 97)		*Empathic-alarmed* (*n *= 75)		*Univariate*	
	*M*	*SD*	*M*	*SD*	*M*	*SD*	*F*	η^2^
Grief	1.25^a^	0.43	2.28^b^	0.90	3.29^c^	1.08	157.81***	.51
Despair	1.25^a^	0.49	2.49^b^	0.84	3.63^c^	1.01	223.42***	.60
Ashamed	1.26^a^	0.48	2.44^b^	0.96	3.80^c^	1.00	240.27***	.61
Hurt	1.28^a^	0.56	2.15^b^	0.99	3.68^c^	0.96	198.45***	.57
Depressed	1.33^a^	0.63	1.92^b^	0.95	3.37^c^	1.09	109.21***	.47
Guilty	1.34^a^	0.62	2.39^b^	0.97	3.69^c^	1.12	154.62***	.53
Angry	1.34^a^	0.56	2.71^b^	0.92	4.04^c^	0.88	307.01***	.66
Disgusted	1.41^a^	0.66	3.00^b^	1.06	4.03^c^	0.90	260.22***	.60
Afraid	1.41^a^	0.62	2.40^b^	0.92	3.96^c^	0.83	261.53***	.63
Confused	1.50^a^	0.77	2.23^b^	1.00	2.93^c^	1.19	48.71***	.26
Anxious	1.50^a^	0.73	2.36^b^	0.84	3.93^c^	0.94	181.60***	.58
Sad	1.62^a^	0.67	3.03^b^	0.88	4.28^c^	0.69	349.61***	.67
Frustrated	1.63^a^	0.73	3.04^b^	1.04	4.13^c^	0.72	273.01***	.59
Doubtful	1.67^a^	0.93	2.36^b^	1.00	3.12^c^	1.17	42.90***	.25
Worried	1.72^a^	0.77	3.00^b^	0.94	4.24^c^	0.59	322.16***	.62
Helpless	1.82^a^	1.04	2.59^b^	0.94	3.97^c^	1.07	93.51***	.42
Powerless	2.15^a^	1.21	2.94^b^	1.14	4.00^c^	1.04	62.95***	.30
Compassionate	2.24^a^	1.07	2.95^b^	1.05	3.51^c^	1.06	33.44***	.19
Bored	1.51^a^	0.91	1.60^a^	0.94	1.91^a^	1.18	3.10	.03
Indifferent	1.77^a^	0.95	1.94^a^	0.97	2.13^a^	1.28	2.35	.02
Happy	1.86^a^	1.14	1.65^a^	1.04	1.71^a^	1.00	1.03	.01
Optimistic	2.18^a^	1.16	2.12^a^	1.05	2.33^a^	1.16	0.77	.01
Hopeful	2.24^a^	1.10	2.28^a^	1.06	2.59^a^	1.23	2.15	.02

*N* = 286. *V* = 1.10, *F*(46, 524) = 13.94, *p* < .001, η^2^ = .55. Means with different superscripts (in rows) differ significantly at *p* < .01 (Gabriel). *** *p* < .001.

In terms of demographic characteristics, the three segments differed significantly only in age. Members of the emotionally-detached (*M* age* *= 48.54, *SD* = 18.04) and emotionally-ambivalent (*M* age = 43.39, *SD* = 17.15) segments were significantly older than the empathic-alarmed (*M* age = 34.56, *SD* = 13.62). Table 4 contains a demographic breakdown of the three retained segments.

**Table 4:** Demographic characteristics of the three eco-emotion segments.

Segment Variables	*Emotionally-detached* (*n *= 114)		*Emotionally-ambivalent* (*n *= 97)		*Empathic-alarmed* (*n *= 75)		*Univariate*	
	*M*	*SD*	*M*	*SD*	*M*	*SD*	*F*	r
Age	48.54^a^	18.04	43.39^a^	17.15	34.56^b^	13.62	18.89***	−0.31
								
	***%***	***Z*_*Resid*_**	***%***	***Z*_*Resid*_**	***%***	***Z*_*Resid*_**	***χ* ^*2*^ *(df)***	***V***
Gender:							8.92(4)	0.12
Male	39	1.07	30	−0.51	28	−0.74		
Female	61	−0.66	70	0.44	69	0.31		
Non-binary	0	−0.89	0	−0.82	3	2.04		
Education:							15.73 (12)	0.14
Lower than secondary school	4	0.75	2	−0.60	3	−0.23		
Secondary school	31	0.86	24	−0.55	24	−0.43		
Trade/ professional/ technical qualification	34	0.95	25	−0.84	28	−0.22		
Undergraduate degree	16	−1.14	28	1.56	19	−0.37		
Honors/ postgraduate degree	10	−0.88	13	0.23	16	0.83		
Master’s degree	4	−0.81	8	0.77	7	0.13		
PhD	1	−0.47	0	−1.16	4	1.90		
Living Area:							10.65 (8)	0.14
Inner City	15	−1.05	21	0.31	24	0.94		
Suburban	43	−0.28	51	0.85	40	−0.62		
Residential in rural Town	18	1.02	12	−0.43	11	−0.77		
Semi-rural	5	−0.57	8	0.61	7	0.01		
Rural	19	1.07	8	−1.79	19	0.72		
								

*N* = 286. Means with different superscripts (in rows) differ significantly at *p* < .01 (Gabriel). *r* = Pearson’s correlation coefficient. *Z*_*Resid *_= adjusted standardized residual. *V* = Cramer’s *V*. *χ*^*2*^ analyses conducted with 10,000 bootstrap samples. *** *p* < .001.

### Segment membership, environmental concern, and willingness to sacrifice

**Fig 2:**
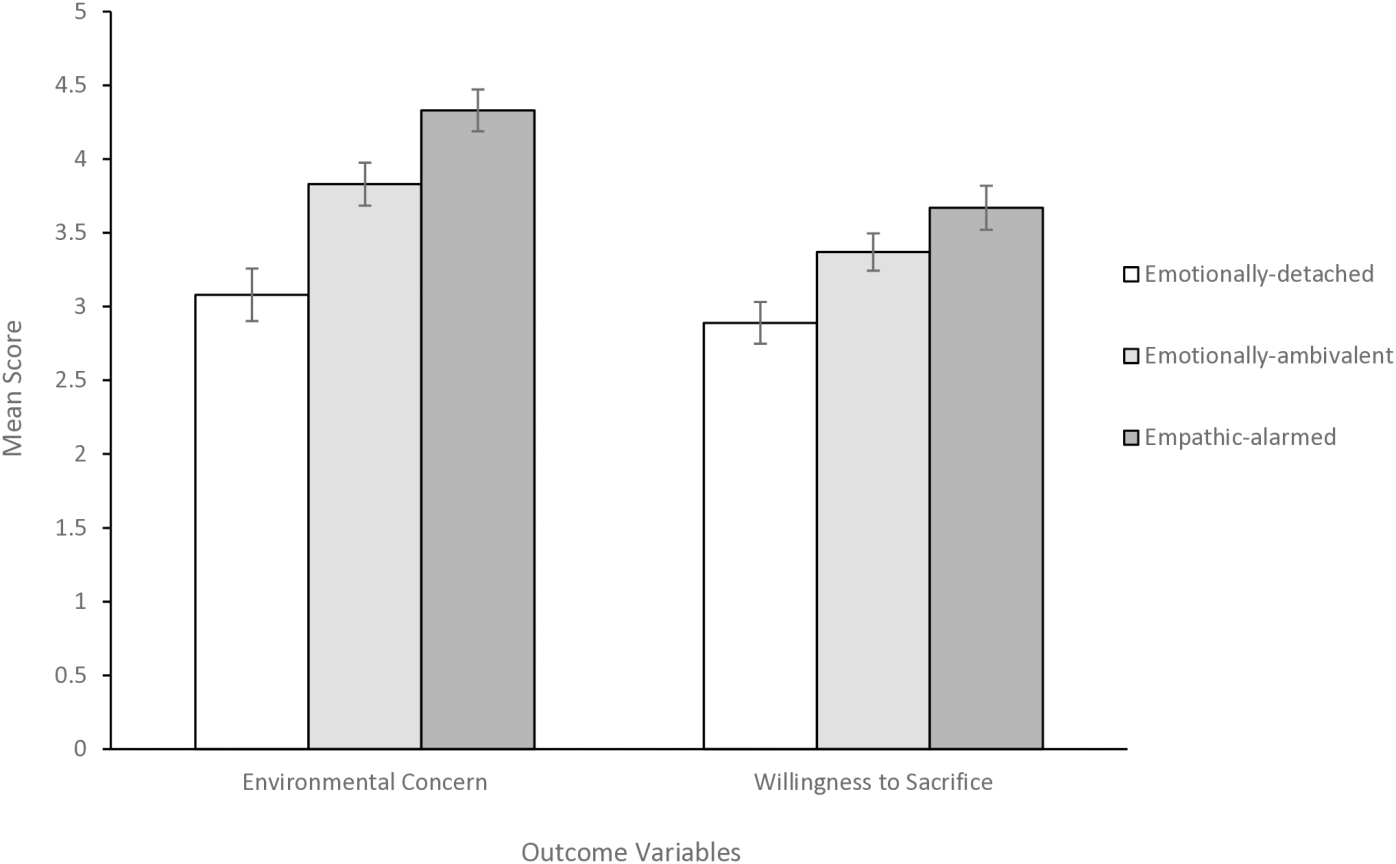
Mean scores for environmental concern and willingness to sacrifice across the three eco-emotion segments. *Notes. N *= 286. Emotionally-detached, *n* = 114. Emotionally-ambivalent, *n* = 97. Empathic-alarmed, *n *= 75. Error bars represent 95% confidence interval. Environmental concern was measured with seven items on a 5-point Likert scale (1 = strongly disagree to 5 = strongly agree). Willingness to sacrifice was measured with 24 items, all measured on a 5-point Likert scale (1 = strongly disagree to 5 = strongly agree).

MANOVA was used to investigate whether the segments differed significantly in their environmental concern and willingness to sacrifice. The MANOVA revealed a significant effect (Λ = 0.69, *F*(4, 566) = 25.93, *p* = .001), with segment membership explaining 31% of the variance in the combined dependent variables (environmental concern and willingness to sacrifice). Two follow-up one-way ANOVAs revealed statistically significant large main effects of group membership on levels of environmental concern*, F*(2, 186.82) = 59.56, *p* < .001, η^2 ^= 29, and willingness to sacrifice*, F*(2, 180.16) = 29.75, *p* < .001, η^2 ^= .18, indicating that the segments differed significantly in their levels of environmental concern and willingness to sacrifice. Results from Gabriel’s post hoc tests revealed that members of the empathic-alarmed segment (*M = *4.33, *SD *= 0.62) had significantly higher levels of environmental concern (*p* < .001) than members of the emotionally-ambivalent (*M = *3.83, *SD *= 0.72) and the emotionally-detached (*M = *3.08, *SD *= 0.96; Fig 2) segments. Moreover, emotionally-ambivalent respondents were significantly more concerned about the environment than emotionally-detached ones (*p* < .001).

For willingness to sacrifice, members of the empathic-alarmed (*M *= 3.67, *SD *= 0.65) and emotionally-ambivalent (*M *= 3.37, *SD *= 0.63) segments scored significantly higher (*p* < .001) than the emotionally-detached segment (*M = *2.89, *SD *= 0.76). Respondents in the empathic-alarmed segment were also significantly more willing to make sacrifices for the environment than the respondents in the emotionally-ambivalent segment (*p* = .015). Overall, these findings support our hypothesis that, based on participants’ emotional responses to environmental problems, several segments exist, and that members of each segment differ significantly in their environmental concern and willingness to sacrifice.

## Discussion

The current study aimed to extend our understanding of which eco-emotions occur together, and how patterns of eco-emotions predict environmental concern and willingness to make sacrifices for the environment. We found that respondents could be segmented into three main profile groups based on their emotional responses to environmental problems. Moreover, our results indicated that group membership predicted both, environmental concern and willingness to make sacrifices for the natural environment. Each of these findings is discussed in more detail in the sections that follow, along with a discussion of practical implications and limitations of the research.

### Eco-emotion profile groups

Applying LPA to 23 eco-emotions, we identified three distinct segments which were characterized as being emotionally-detached (40%), emotionally-ambivalent (34%), or empathic-alarmed (26%) about environmental problems such as climate change. Contrary to previous studies [18,32,33,50], we found that the segments differed only in their levels of negative eco-emotions and compassion, but not in their positive (i.e., optimism, hope, happiness) and apathetic eco-emotions (i.e., indifference, boredom). The largest segment, the emotionally-detached, reported lower levels of all negative eco-emotions and compassion than the emotionally-ambivalent who in turn reported less intense negative eco-emotions and compassion than the smallest segment, the empathic-alarmed. As the three segments only differed in their negative eco-emotions and compassion, but not in their positive and apathetic eco-emotions, hypothesis 1 was only partially supported.

In terms of their socio-demographic characteristics, the three segments differed in age, but not in gender, education, and living area. The empathic-alarmed were younger than the emotionally-ambivalent and the emotionally-detached. This aligns with previous studies which found that younger generations tend to be more engaged and emotionally involved with environmental problems [51] than older generations (e.g., feeling more anxious, angry, and less annoyed when shown climate change related social media content).

The identified segments have some similarities with those reported by Wang and colleagues [18] who surveyed Australian residents, but also some key differences. Wang and colleagues identified four segments, which differed in both their intensities and valence of eco-emotions. Their segments were characterized as either feeling strong negative emotions (e.g., anger and fear), weak negative emotions (e.g., anger and fear experienced with lower intensity), ambivalent emotions (moderate levels of positive and negative emotions), or no emotions (low levels of positive and negative emotions). In contrast to their segmentation, we identified three segments, which differed only in their intensity of experienced eco-emotions. Using a different statistical approach may have contributed to these findings. While Wang and colleagues used hierarchical cluster analysis to identify emotion profiles within their sample, we employed LPA, which is considered the gold standard for segmenting populations and allowed us to use fit indices to identify a segment solution which best fits the data [52,53]. Moreover, while Wang and colleagues reported differences between the four segments in positive (i.e., hopeful, joyful, excited), apathetic (i.e., bored), and negative eco-emotions (e.g., angry, irritated), the segments in the current study only differed in their negative eco-emotions and compassion. The emotionally-detached segment in the current study was similar to the no emotion segment identified by Wang and colleagues, in that they both experienced low levels of all eco-emotions. However, the no-emotion segment (24%) they identified was only around half the size of the emotionally-detached segment found in the current study (40%). This suggests a potential cross-cultural difference, with a higher proportion of New Zealanders compared to Australians being emotionally-detached with environmental problems. One explanation for this could be that Australians are more directly impacted by environmental problems such as climate change than New Zealanders [54], resulting in Australians having less psychological distance towards environmental issues [26], which in turn may have led to a greater emotional response [55].

### Emotion patterns predict environmental concern and willingness to sacrifice

As hypothesized, segment membership predicted how environmentally concerned and willing to make sacrifices the segments were. All segments experienced different levels of environmental concern, with the empathic-alarmed being the most concerned, followed by the emotionally-ambivalent and the emotionally-detached. In terms of willingness to sacrifice, individuals who were empathic-alarmed were more willing to give up unsustainable behaviors than emotionally-ambivalent individuals, who in turn were more willing to do so than those in the emotionally-detached segment. The stronger negative eco-emotions felt by the empathic-alarmed and emotionally-ambivalent may have made the risks associated with environmental problems be perceived as less abstract and thus concrete and salient [6,9,56,57], resulting in greater environmental concern and willingness to make sacrifices. Overall, our findings align broadly with those of previous research, in that emotions are important in shaping pro-environmental attitudes and decision-making processes. Our study supports previous research that suggests eco-emotions may be critical to be concerned about environmental problems [25], to be willing to make sacrifices for the natural world [36], and to perform PEB [17,18]. Experiencing eco-emotions may have helped to concretize abstract environmental problems and in turn increased the motivation to perform PEB [9,29,56–59].

A difference to previous findings [17,18,60] was that the three segments identified in this study differed primarily in their experienced magnitude of negative eco-emotions and compassion. This suggests that a combination of these types of eco-emotions sets groups of people apart from each other. The importance of negative eco-emotions aligns with findings of a meta-analysis by van Valkengoed and Steg [61], who found that together with outcome efficacy and descriptive norms, negative affect was a strong predictor for a range of climate change adaptation behaviors such as policy support. The unpleasantness of a negative emotional arousal caused by experiencing a range of negative emotions may have acted as a motivator to increase intentions to act environmentally-friendly [62].

In addition to differences in all negative emotions between the three segments, members of the emotionally-detached, emotionally-ambivalent, and empathic-alarmed segments differed in their levels of compassion, a finding that contributes to previous research in this area [63,64]. In a recent study, Pfattheicher and colleagues [63] found that participants who were assigned to a high-compassionate condition (i.e., had to imagine how a person feels) had higher pro-environmental intentions than those assigned to a low-compassionate condition. Similar results were reported by Engel and colleagues [64] who found that entrepreneurs who listened to a guided loving-kindness meditation had higher levels of compassion and made more sustainable decisions than a group which only listened to a talk about meditation (without actually meditating). In their study, the relationship between the loving-kindness meditation and pro-environmental decision making was mediated by compassion. In the current study, compassion, which evokes a feeling for the suffering of others [65], may have increased the awareness of how one’s actions impact others negatively, reduced psychological distance, and elicited moral concern [26,63,64]. This in turn may have acted as a motivator for alleviating the suffering of others and hence may have contributed to the willingness to practice PEB.

In line with Ajzen’s TPB [23], Slovic and colleagues’ affect heuristic model [11,24], and research on psychological distance [26] our findings suggest that eco-emotions may play an important role in shaping motivational pathways underlying PEB.

### Practical implications

The results of the current study support the view that emotions may be critical for guiding judgements and decisions related to PEB. The fact that the three segments differed primarily in terms of the relative intensity with which segment members experienced negative eco-emotions and compassion can help in the development of communication interventions, which evoke specific sets of emotions (i.e., by inducing a combination of negative emotions and compassion) to engage with groups of people more effectively.

Our results contribute to findings from a growing body of research on the effects of emotions on PEB. Invoking specific negative emotions such as guilt [28,30,31,36,66–68], fear [28,36,69], frustration, anger and depression [34], grief [31,68], and worry [29] have previously all been found to promote PEB, while experiencing sadness and shame on their own were found to be associated with decreased levels of PEB [70]. Mixed effects of anxiety on PEB were previously reported [31,34,71–73]. The current study found that all negative emotions (including sadness, shame, and anxiety) were higher in groups that reported greater levels of environmental concern and willingness to sacrifice. Thus, it may be that the interaction of negative eco-emotions and compassion increases concern for the environment and intentions to sacrifice unsustainable behaviors. In order for individuals to shift to a segment that is more emotionally engaged and hence more motivated to make sacrifices to benefit the natural environment, it may be beneficial to induce a combination of negative eco-emotions and compassion.

To further support the success of interventions eliciting specific patterns of eco-emotions, additional factors may need to be considered, including the level of self-efficacy [27,74], ethical considerations [31,34,71,75], and potential adverse effects of too intensely experienced eco-emotions [76]. Self-efficacy has previously been suggested to influence the course of action. According to the extended parallel process model [74], when facing a threat, experiencing negative emotions such as fear only leads to action when a high level of self-efficacy is present. However, when self-efficacy is low, individuals may deny the threat or actively react against the threat (e.g., “climate change is not real”) in order to manage their distressing emotions. Hence, to increase the success of emotion-inducing interventions it may be advisable to integrate a self-efficacy element [27]. One way to achieve this is by providing information on how to decrease unsustainable behaviors. This in turn may increase confidence that one’s actions contribute to the planet’s health and therefore, lead to PEB. A second point to consider is the intensity of experienced eco-emotions. While the current study did not find a reversal effect of stronger experienced eco-emotions on environmental concern and willingness to sacrifice, Pihkala [76] suggests that when the intensity of negative eco-emotions (e.g., climate anxiety) is too high, it may paralyze people and thereby lead to inaction. Moreover, when negative eco-emotions are too strong, they were previously found to be associated with detrimental mental health outcomes and increased levels of insomnia [34,75]. Therefore, from an ethical point of view, it is important to develop emotion-inducing interventions with a safe level of intensity, which in turn may also contribute to an intervention’s success.

In summary, developing interventions that boost compassion and negative eco-emotions (within a safe range) may be a promising strategy to increase PEB in emotionally-detached and emotionally-ambivalent individuals. This approach may be particularly impactful for samples of respondents like ours in which the emotionally-detached and emotionally-ambivalent segments comprised nearly three quarters of all participants surveyed.

### Limitations and future research

When interpreting our findings, several limitations have to be considered. First, we surveyed a broad range of New Zealand residents. In terms of age and ethnicity the current sample was broadly representative of the New Zealand population, although females were over-represented. In addition, our reliance on an online panel may have excluded individuals who either lack internet access or the motivation and skill required to navigate online surveys [77].

Second, our study used a cross-sectional correlational methodology assessing a limited range of variables. Although finding that empathic-alarmed participants were more willing to make sacrifices for the environment suggests one possible path forward for increasing public engagement with environmental issues, our research design does not permit causal inferences from being drawn from our data. Experimental research designed to elicit complex motivating patterns of emotional responses (e.g., as displayed by our empathic-alarmed profile) are necessary to establish causality. It is also important to acknowledge that the path from eco-emotions to behavior is likely far more complex, with a range of potential moderating and mediating factors not modeled in this study.

Third, our study relied on self-report measures which are prone to socially desirable responding [78] and may have affected the validity of this study. To mitigate this risk, participants were recruited through a third-party, the study was conducted online, and information provided was kept anonymous. Relatedly, given that measuring PEB in real world settings is often difficult, we used willingness to sacrifice as a self-report proxy measure in this study. This allowed us to include a wide range of sustainable behaviors and hence have a more holistic picture of participants’ pro-environmental intentions. While there may be a gap between self-reported willingness to sacrifice and actual behavior, research suggests that intentions are often the strongest predictor of behavior [79]. Intentions that involve sacrifice may be particularly predictive given they suggest a willingness to engage in behaviors despite the barriers that may be present.

Future research can extend and validate the findings of our study by investigating whether messages which induce a combination of negative emotions and compassion simultaneously are more effective in motivating viewers than messages that evoke a combination of other sets of eco-emotions. Experiments with behavioral outcomes, as opposed to proxy measures like willingness to sacrifice, will be particularly valuable in improving our understanding of the potential causal impact of how message content and framing can be used to elicit patterns of eco-emotions conducive to PEB. Finally, further quantitative survey research with a broader range of psychological variables, as well as qualitative research focusing on personal phenomenological experiences of emotional responses, would be beneficial to develop a deeper and more nuanced understanding of how complex combinations of eco-emotions shape perceptions, motivations, decisions, and behaviors related to protecting the environment.

## Conclusion

Overall, our findings indicate that eco-emotion profiles reliably predict environmental concern and willingness to make personal sacrifices to protect the environment. We identified three eco-emotion segments in our New Zealand sample (emotionally-detached, emotionally-ambivalent, and empathic-alarmed). Members of the three segments differed in the degree to which they experienced negative eco-emotions and compassion, but not in their experiences of other positive eco-emotions and apathy. Members of the empathic-alarmed segment expressed more concern about the environment and were more willing to make sacrifices for it than members of the emotionally-ambivalent segment, who in turn were more concerned about the environment and more willing to make sacrifices to protect it than members of the emotionally-detached segment. Targeting the two largest segments (emotionally-detached and emotionally-ambivalent) with appeals that focus on eliciting negative emotions alongside compassion may prove to be an effective way to increase pro-environmental behavior in these groups. These results underscore the value of using segmentation approaches to identify the common patterns of eco-emotions people experience and how these patterns shape motivations to save the planet.

## Supporting information

S1 Table Demographic characteristics. (DOCX)

S2 Appendix Questionnaire. (DOCX)
