# Supplementary material for: From detached to alarmed: How eco-emotion profiles predict concern and sacrifice for the planet
Source: PLoS One. 2025 Jun 17;20(6):e0325916. doi: 10.1371/journal.pone.0325916 (PMC12173424; doi:10.1371/journal.pone.0325916)
Supplement: S1 Table — (DOCX) [file pone.0325916.s001.docx]

**S1 Table. Demographic characteristics**

| Variable | Sample |
| --- | --- |
|  |  |
| Age (in years) |  |
| M (SD) | 43.13 (17.53) |
| Median | 40.00 |
|  |  |
| Gender (%) |  |
| Male | 33 |
| Female | 66 |
| Non-binary | 1 |
|  |  |
| Ethnicity (%) |  |
| New Zealand European / Pākehā | 67 |
| Māori | 17 |
| Chinese | 3 |
| Others | 13 |
|  |  |
| Highest Qualification (%) |  |
| Lower than secondary school | 3 |
| Secondary school | 27 |
| Trade / technical / professional  qualification | 29 |
| Undergraduate Degree | 21 |
| Honors Degree or Postgraduate Certificate | 13 |
| Master’s Degree | 6 |
| PhD | 1 |
|  |  |
| Living Area (%) |  |
| Inner City | 19 |
| Suburban | 45 |
| Residential in rural town | 14 |
| Semi-rural | 7 |
| Rural | 15 |
|  |  |

*Note.* Participants could choose more than one ethnicity. Ethnicity “Others” included Samoan (1%), Cook Island Māori (1%), Tongan (1%), Indian (1%).
