# Supplementary material for: From detached to alarmed: How eco-emotion profiles predict concern and sacrifice for the planet
Source: PLoS One. 2025 Jun 17;20(6):e0325916. doi: 10.1371/journal.pone.0325916 (PMC12173424; doi:10.1371/journal.pone.0325916)
Supplement: S2 Appendix — (DOCX) [file pone.0325916.s002.docx]

S2 Appendix. Questionnaire

***Start of Survey***

Before you proceed with the survey, please complete the captcha below

***Informed Consent & Welcome***

Welcome to the research study!

We are interested in better understanding Kiwis' feelings, beliefs, and behaviours related to environmental problems such as climate change.

The questionnaire should take you around 10-15 minutes to complete. Your participation in this research is voluntary. You have the right to withdraw at any point during the study. All your responses will be completely anonymous.

The Principal Investigator of this study is Andreas K Jaeger and can be contacted at aja223@uclive.ac.nz.

This project has been approved by the Educational Research Human Ethics Committee (human-ethics@canterbury.ac.nz).

Although this questionnaire is unlikely to raise any personal or upsetting issues, if it does you may wish to contact Lifeline on 0800 543 354 or your GP.

By clicking the button below, you acknowledge:
Your participation in this research is voluntary.
You are 18 years of age or over.
You agree to participate in this study and are aware that you may choose to withdraw at any time for any reason.

***Sociodemographic***

These first questions are to learn something about you

What is your gender?

- Male
- Female
- Non-binary / third gender
- Prefer not to say

What ethnic group do you belong to? Select all that apply to you.

- New Zealand European / Pākehā
- Māori
- Samoan
- Cook Islands Māori
- Tongan
- Niuean
- Chinese
- Indian
- Other (please specify) __________________________________________________

What is the highest qualification you have received?

- Less than secondary school
- Secondary school qualification
- Trade / technical certificate or professional qualification
- Undergraduate qualification (Bachelors degree or Undergraduate Diploma)
- Bachelor Honours or Postgraduate Certificate, Diploma or Degree
- Masters degree
- PhD

Which of the following best describes the area where you live?

- Inner city
- Suburban
- Residential in rural town
- Semi-rural / Peri-urban (e.g., acreage on the edge of town)
- Rural

What is your age?

- 18
- 19
- 20

(up to 100)

***Eco-emotions, Environmental Concern & Willingness to Sacrifice***

For each of the words below, indicate how environmental problems such as climate change make you feel.

Please rate how much you agree with the following statements.

|  | Not at All | Slightly | Moderately | Very | Extremely |
| --- | --- | --- | --- | --- | --- |
| Sad |  |  |  |  |  |
| Helpless |  |  |  |  |  |
| Anxious |  |  |  |  |  |
| Afraid |  |  |  |  |  |
| Optimistic |  |  |  |  |  |
| Angry |  |  |  |  |  |
| Guilty |  |  |  |  |  |
| Ashamed |  |  |  |  |  |
| Hurt |  |  |  |  |  |
| Depressed |  |  |  |  |  |
| Despair |  |  |  |  |  |
| Grief |  |  |  |  |  |
| Powerless |  |  |  |  |  |
| Indifferent |  |  |  |  |  |
| Hopeful |  |  |  |  |  |
| Disgust |  |  |  |  |  |
| Happy |  |  |  |  |  |
| Compassionate |  |  |  |  |  |
| Confusion |  |  |  |  |  |
| Boredom |  |  |  |  |  |
| Doubt |  |  |  |  |  |
| Worry |  |  |  |  |  |
| Frustration |  |  |  |  |  |

|  | Strongly disagree | Somewhat disagree | Neutral | Somewhat agree | Strongly agree |
| --- | --- | --- | --- | --- | --- |
| I am very concerned about environmental problems such as climate change |  |  |  |  |  |
| I am very concerned about potential impacts of environmental problems such as climate change that may affect me personally. |  |  |  |  |  |
| I am very concerned about potential impacts of environmental problems such as climate change that may affect society in general. |  |  |  |  |  |
| I experience concern each time I see or read media coverage of the likely impacts and consequences of environmental problems such as climate change. |  |  |  |  |  |
| The more I learn about environmental problems such as climate change, the more concerned I become. |  |  |  |  |  |
| I am not overly concerned about environmental problems such as climate change as I think the impacts are probably exaggerated. |  |  |  |  |  |
| I am concerned that the impacts of environmental problems such as climate change will affect me in the foreseeable future. |  |  |  |  |  |

According to scientists, human behaviour is causing environmental problems such as climate change.

Please indicate on the scale below how willing you are to make changes to your behaviour to prevent environmental problems from happening.

|  | Strongly disagree | Mildly disagree | Neither disagree nor agree | Mildly agree | Strongly agree |
| --- | --- | --- | --- | --- | --- |
| I am willing to buy an electric car rather than a fossil-fuel car, even if the electric car is more expensive |  |  |  |  |  |
| To reduce my environmental impact, I often walk, cycle, or use public transport instead of driving my car to nearby places |  |  |  |  |  |
| I try to avoid travelling within NZ by plane, even if travelling by plane is most convenient |  |  |  |  |  |
| I am willing to pay more for my flights to offset the carbon emissions produced through my flight |  |  |  |  |  |
| I am willing to reduce my overseas travel by at least 50% |  |  |  |  |  |
| I am willing to adopt a meat-free diet |  |  |  |  |  |
| I am willing to reduce my meat consumption by 50% to reduce my greenhouse gas footprint |  |  |  |  |  |
| I am willing to use milk-alternatives instead of dairy milk |  |  |  |  |  |
| To show that you have read this sentence, in this line we ask you to mark 'Strongly agree' on the answer scale |  |  |  |  |  |
| I am willing to make an extra effort to find and buy locally produced food |  |  |  |  |  |
| I am willing to pay more for food that is environmentally-friendly |  |  |  |  |  |
| I am willing to compost food scraps instead of throwing them in the rubbish |  |  |  |  |  |
| I am willing to buy electric devices that have a better energy efficiency rating, even if they are more expensive than less energy efficient options |  |  |  |  |  |
| I am willing to pay more for electricity that comes renewable sources |  |  |  |  |  |
| In winter, I often put on an extra layer of clothes rather than turning the heater on |  |  |  |  |  |
| I am willing to buy second-hand products over brand-new products |  |  |  |  |  |
| I am willing to avoid products with much packaging, even if they are more expensive |  |  |  |  |  |
| I avoid single-use products (e.g., straws, takeaway coffee cups) even though they are convenient |  |  |  |  |  |
| I am planning not to have any children |  |  |  |  |  |
| I am willing to pay higher taxes for conservation efforts in New Zealand |  |  |  |  |  |
| I generally try to buy new furniture where the wood is sustainably sourced, even when those products are more expensive |  |  |  |  |  |
| I am willing to accept less pay to work for a company/organization that has strong “green” policies and procedures |  |  |  |  |  |
| I am willing to take shorter showers to conserve water |  |  |  |  |  |
| I am willing to spend most of my holidays in New Zealand instead of going overseas by plane |  |  |  |  |  |
| I support paying more money for clothes that are sustainable (made of natural fibres or recycled materials) |  |  |  |  |  |
